# Positively-Charged Semi-Tunnel Is a Structural and Surface Characteristic of Polyphosphate-Binding Proteins: An In-Silico Study

**DOI:** 10.1371/journal.pone.0123713

**Published:** 2015-04-16

**Authors:** Zheng Zachory Wei, Greg Vatcher, Alvin Hok Yan Tin, Jun Lin Teng, Juan Wang, Qing Hua Cui, Jian Guo Chen, Albert Cheung Hoi Yu

**Affiliations:** 1 Neuroscience Research Institute, Peking University; Department of Neurobiology, School of Basic Medical Sciences, Peking University; Key Laboratory for Neuroscience (Peking University), Ministry of Education; Key Laboratory for Neuroscience (Peking University), National Health and Family Planning Commission, Beijing 100191, China; 2 The Key Laboratory of Cell Proliferation and Differentiation of Ministry of Education, The State Key Laboratory of Bio-membrane and Membrane Bio-engineering, College of Life Sciences, Peking University, Beijing 100871, China; 3 Department of Medical Informatics, School of Basic Medical Sciences, Peking University, Beijing 100191, China; 4 Infectious Disease Center, Peking University, Beijing 100191, China; 5 Laboratory of Translational Medicine, Institute of Systems Biomedicine, Peking University, Beijing 100191, China; Russian Academy of Sciences, Institute for Biological Instrumentation, RUSSIAN FEDERATION

## Abstract

Phosphate is essential for all major life processes, especially energy metabolism and signal transduction. A linear phosphate polymer, polyphosphate (polyP), linked by high-energy phosphoanhydride bonds, can interact with various proteins, playing important roles as an energy source and regulatory factor. However, polyP-binding structures are largely unknown. Here we proposed a putative polyP binding site, a positively-charged semi-tunnel (PCST), identified by surface electrostatics analyses in polyP kinases (PPKs) and many other polyP-related proteins. We found that the PCSTs in varied proteins were folded in different secondary structure compositions. Molecular docking calculations revealed a significant value for binding affinity to polyP in PCST-containing proteins. Utilizing the PCST identified in the β subunit of PPK3, we predicted the potential polyP-binding domain of PPK3. The discovery of this feature facilitates future searches for polyP-binding proteins and discovery of the mechanisms for polyP-binding activities. This should greatly enhance the understanding of the many physiological functions of protein-bound polyP and the involvement of polyP and polyP-binding proteins in various human diseases.

## Introduction

The phosphate form of phosphorus (PO_4_^3-^) is essential for all life and cellular processes, especially energy metabolism and signal transduction. Polymers of phosphates, polyphosphate (polyP), discovered in 1888 [1], contain a few to several hundred residues of orthophosphate linked by high-energy phosphoanhydride bonds. PolyP has since been documented in mammalian cells [2], and has been revealed to have physiological roles [3], including regulation of vertebrate skeletal mineralization [4–7], fibrin clotting [8–10], intestinal homeostasis [11], neuronal excitability [12], innate immunity [13] and to have functions as a P2 receptor agonist [14]. PolyP is also involved in some disease conditions, including tumor proliferation [15,16], metastasis [17], cardiac necrosis or neurodegenerative processes [17–20] and energy deficiency [21–23].

The important physiological functions of polyP are potentially regulated via protein binding [24–30] and the interaction between polyP and polyP-related proteins (enzymes or regulatory proteins) are functionally relevant to these roles, however, polyP-interacting sequences or structures are largely unknown. The reported polyP-related proteins show distinct and controversial structural features for polyP-binding [31–37]. The deep S-shaped canyon in exopolyphosphatase (PPX) from *Escherichia coli* contains the active site region [38]. Another PPX from *Saccharomyces cerevisiae* has a channel representing a conduit for polyP [35]. In glucomannokinase (GMK), the cleft between two domains is a potential polyP-binding site [39]. Although there are large differences in the overall structures of known polyP binding proteins, we hypothesized in this study that the polyP-binding regions in related proteins (both enzymes and regulatory proteins) displayed a tunnel-shaped structure.

We investigated this tunnel in polyP-related proteins (both enzymes and regulatory proteins), and proposed the positively-charged semi-tunnel (PCST) as the common polyP binding domain. Surface electrostatics analyses revealed positive charges at tunnel surfaces in the four known PPK families, groups of enzymes that catalyze the elongation and synthesis of polyP [40–43]. Sequence comparisons and structural superimpositions revealed that the PCST in different proteins were folded in different secondary structure compositions. Molecular docking calculations using short-chain polyP-containing ligands showed significant correlation between predictive “polyP-binding” and “PCST-containing” proteins. Our observed protein surface and feature structure was a characteristic of polyP-binding proteins and a potential binding site for polyP. This study presents interesting insights and methodology for future searches for polyP-binding proteins.

## Materials and Methods

### Sequence analyses

Sequences of PPKs were retrieved from the UniProt Knowledge Base (www.uniprot.org). Basic Local Alignment Search Tool (BLAST) and Translated BLAST (TBLASTN) [44] were applied for searching homologous entries. Homologous genes were screened manually from the NCBI HomoloGene resource (www.ncbi.nlm.nih.gov/homologene) or a BLAST search following multiple sequence alignment. Homologous sequences were aligned using CLUSTALW or the MUSCLE program integrated in the MEGA software [45], followed by manual correction for several amino acid positions. We also employed alignment tools in the WebLab platform [46] and TCOFFEE [47] for structure-aided sequence analyses.

### Structural comparison

Structures were retrieved from the Research Collaboratory for Structural Bioinformatics (RCSB) Protein Data Bank (PDB) web site (www.rcsb.org) and enzymatic molecular structures were visualized and compared using the Swiss-PDB Viewer [48] (www.expasy.org/spdbv) or PyMOL (www.pymol.org). Structural alignment was performed by the Combinatorial Extension (CE) [49] and Dali [50] programs. Z-scores were also calculated to measure the statistical significance of the results relative to the alignment of random structures. A structure comparison method based on protein structures available in the PDB was used for the search. The Dali program was utilized to search all PDB protein structures for those with similarity to PPKs (S1 Text).

### Molecular docking calculations

Protein modeling was based on both 3D-JIGSAW [51] and the Protein Homology /analogY Recognition Engine (PHYRE) [52]. Potential binding affinity was calculated as suggested by the Docking Server (www.dockingserver.com). In the preparation of the proteins, the center of mass of the subunits was selected for the simulation box setup. The files of the polyP-containing organic ligands were downloaded from PubChem (pubchem.ncbi.nlm.nih.gov). The Merck Molecular Force Field 94 (MMFF94) [53] was used for energy minimization. *Gasteiger* partial charges [54] or PM6 semi empirical charges [55] were added to the ligand atoms. AutoDock tools and the Autogrid program integrated in the Docking Server were also used automatically. Each docking experiment was derived from 100 different runs that were set to terminate after 2,500,000 energy evaluations (some used 10 different runs and a maximum of 250,000 energy evaluations). The potential interactions were manually examined in PyMOL (www.pymol.org).

### 3D visualization and surface electrostatics analyses

Structures retrieved from the RCSB PDB database were saved and trimmed into single chain or specific domains. The trimmed structure files were opened with PyMOL. Local protein contact potential was selected for vacuum electrostatics generation for each structure. The default setup was utilized for the visualization ranging from red color (representing acidic charges) to blue color (representing basic charges). Careful examinations were performed by analyzing related structures from the PDB database and comparing the predictive vacuum electrostatics for different chains and domains of a protein. Screenshots were generated in this software.

### Analysis of modeled structures

PPK3 and actin sequences retrieved from UniProt KB were aligned considering the secondary structure revealed by the actin structure. We compared these characteristics manually after obtaining the predicted 3D structures from the 3D-JIGSAW and PHYRE servers. The structure files were saved and opened with PyMOL for structural comparisons. The same methods were applied to generate the vacuum electrostatics for each modeled subunit.

### Statistical analysis

To analyze the results from molecular docking calculations, Graph Prism version 5.0 was used to make graphs and to perform statistical analysis. For polyP positive control proteins and PPK3 subunits, every value from each running result was plotted as a single point. For PPK partial structural analogous, the lowest value among all running results was plotted as a single point. For comparisons between 2 groups, we used the Student 2-tailed t-test. Blind analysis for the identification of PCSTs was performed using PyMOL. Significance was assumed at a P value of 0.05 in all statistical analyses. Randomization was performed, and the sample size was further determined using Power analysis (Power and Precision 4; Biostat, Inc, Englewood, NJ, USA).

## Results

### Identification of the Positively-Charged Semi-Tunnel (PCST) structure

We examined tertiary structures of PPK families (Table 1) and analyzed the surface electrostatics of the proteins. We found that the reported nucleotide binding domain of PPK1 was positioned in a tunnel area, that a semi-tunnel in PPK2 containing large positive charges was reported and that the tunnel-shaped conformation found in VTC4 (PPK4) contained many basic amino acid residues positioned to interact with the phosphate polymer.

**Table 1:** Summary of polyP kinases and their characteristics.

Family of PPKs	Source	Functions	Sequence similarities	Structural characteristics	Accession No.
**PPK1 (PF02503)**	*Escherichia coli* (NP_416996.1, P0A7B1);*Porphyromonas gingivalis* (NP_905970.1, Q7MTR1);*Dictyostelium discoideum* (XP_629002.1, Q54BM7)	conversion of nucleoside diphosphates to nucleoside triphosphates; GDP to ppGpp.	one region partially similar to PLD phosphodiesterase domain, including conserved His435 autophosphorylation site (IPR003414).	four structural domains (N, H, C1, C2) forming a tunnel in the central active site, with an ATP-binding pocket and accommodation for the translocation ofsynthesized polyP.	1XDO, 1XDP, 2O8R
**PPK2 (PF03976)**	*Pseudomonas aeruginosa* (NP_252145.1, Q9HYF1);*Sinorhizobium meliloti* (NP_384613.1 Q92SA6)	conversion of GDP to GTP, or GMP to GDP.	one or two-fused PPK2 domains (IPR005660), including the Walker A motif (GXXXXGK) and the Walker B motif.	a 3-layer α/β/α sandwich fold with an α-helical lid, comprising an extended positively charged patch possibly involved in the binding polyP.	3CZP, 3CZQ, 3RHF
**PPK3 (PF00022)**	*Dictyostelium discoideum* (XP_636500.1, Q54I79; XP_645275.1, O96621; XP_, Q54HE9)	actin-like fiber concurrent with the synthesis of a polyP chain.	actin-related protein families with ATP/ gelsolin/ profilin binding sites (IPR004000).	three-subunit components with actin-like structure.	not reported
**PPK4 (vacuolar transporter chaperone, VTC4)**	*Saccharomyces cerevisiae* (NP_012522.2, P47075)	membrane transport and vesicular traffic.	one SPX domain (IPR004331) at the N terminus, DUF202 (IPR003807) and VTC domain (IPR018966).	polyP-winding tunnel-shaped pocket with nucleotide-and phosphate-binding structures, formed mainly by antiparallel strands with the tunnel walls lined by conserved basic residues.	3G3Q, 3G3R, 3G3T, 3G3U

The table is based on previous published data and bioinformatics databases as indicated in the Table. Accession numbers are from bioinformatics databases or the Enzyme Commission Database of the ExPASy Proteomics Server (EC numbers). For further information on the enzymatic domains and sequences, refer to the UniProt Knowledge Base, the NCBI RefSeq Protein Database, the Pfam Database and the Interpro Database.

**Fig 1:**
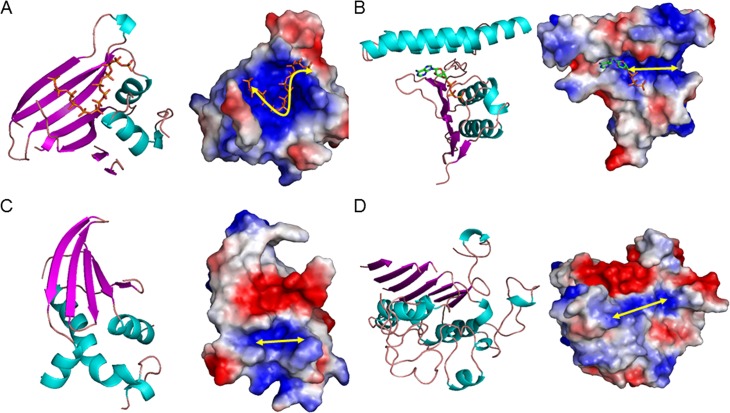
Model structures of Positively-Charged Semi-Tunnel (PCST). (A) PPK4 (PDB ID: 3G3Q); (B) PPK1 (PDB ID: 1XDP); (C) PPX/GPPA (PDB ID: 1T6C); (D) *Hs*PLAP (PDB ID: 1EW2). Protein structure (left) and protein surface graphic (right). Colors on graphics represent surface charges of the 3D structure of the protein. Blue = positive; Red = negative. Yellow double end arrows indicate observed strip of PCST.

By surface electrostatics analyses, we observed the positively-charged area within the surfaces of many other polyP-related proteins (both enzymes and regulatory proteins) where positively-charged amino acid residues constituted a tunnel or semi-tunnel structure. We defined this structure as a “positively-charged semi-tunnel” and potentially identified other proteins that contained PCSTs (S1 Table with their sequence and functional characteristics). We noticed that the PCST was long enough for at least four linearly-linked phosphate groups and that the inside diameter was able to accommodate the phosphate group (Fig 1A).

The secondary structure compositions of the various PCSTs were compared, revealing the diverse nature of the PCST. In PPK4, structures of strands were involved in binding the phosphate groups, while in PPK1, structures of loops and helices might be involved (Fig 1B). The data supported the notion that the structural characteristics of the PCST might contribute to the variable polyP-binding affinity of different PCST-containing proteins.

### The polyP-binding capability of PCST-containing proteins

**Fig 2:**
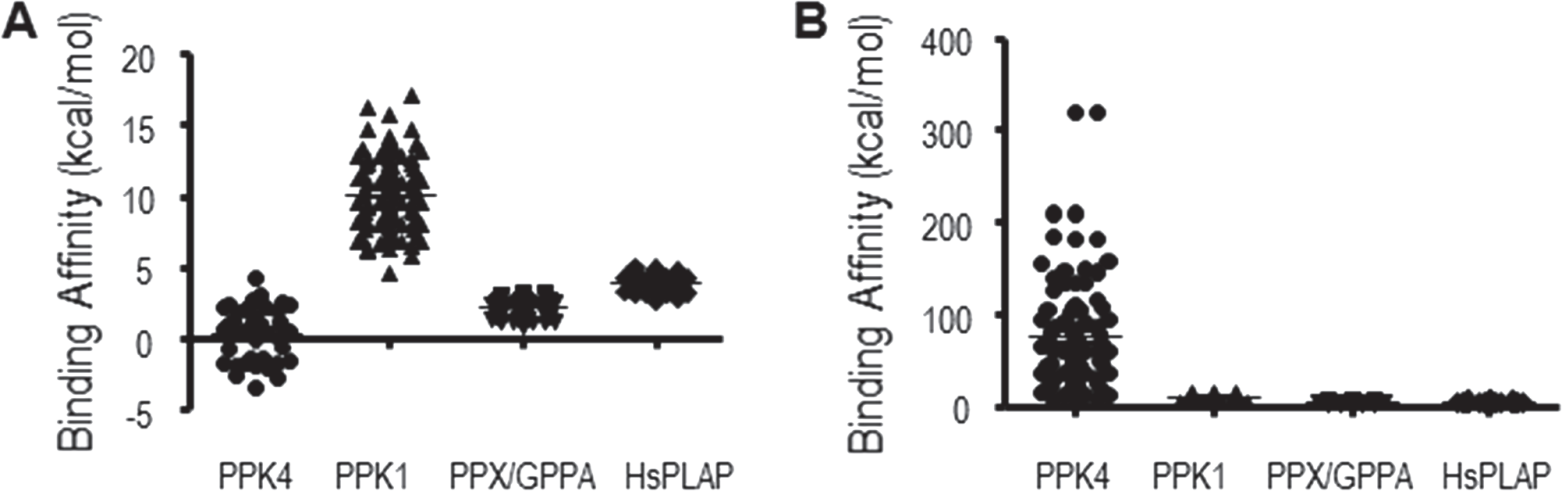
Binding affinities of polyP to polyP positive control proteins. PPK4 (PDB ID: 3G3Q); PPK1 (PDB ID:1XDO);PPX/GPPA (PDB ID: 1T6C); *Hs*PLAP (PDB ID: 1EW2). PolyP ligands included Ap_5_ (A) and Ap_6_A (B). Each circle, square, or triangle represented an estimated free energy of binding from the molecular docking calculations using Ap_5_ or Ap_6_A ligand and different polyP positive control proteins.

To estimate binding affinities of polyP for PCST-containing proteins, molecular docking calculations were performed. Short-chain polyP-containing ligands were used and their binding potentials were reflected by their minimum binding energies. Nucleotide ligands, distinct from short-chain polyP-containing ligands, were also used to confirm the binding of nucleotide. For most calculations, ATP, adenosine pentaphosphate (Ap_5_), or diadenosine hexaphosphate (Ap_6_A) were selected as representatives of nucleotide and polyP, respectively. We first utilized polyP-synthesizing enzymes and polyP-degrading enzymes as positive controls for molecular docking calculations. These proteins included prokaryotic PPK1, yeast PPK4, *Aquifex aeolicus* PPX/GPPA (Fig 1C) and human alkaline phosphatase (Fig 1D), whose structures were all revealed to contain PCST. The blind docking calculations were performed and all the calculated binding free energies were analyzed together (Fig 2). The binding affinities for the positive control proteins were high in all Ap_5_-based and some Ap_6_A (PPK1, PPX/GPPA and HsPLAP)-based dockings. We also noticed that the minimum binding free energies were lower than 100 kcal/mol for these positive controls.

**Fig 3:**
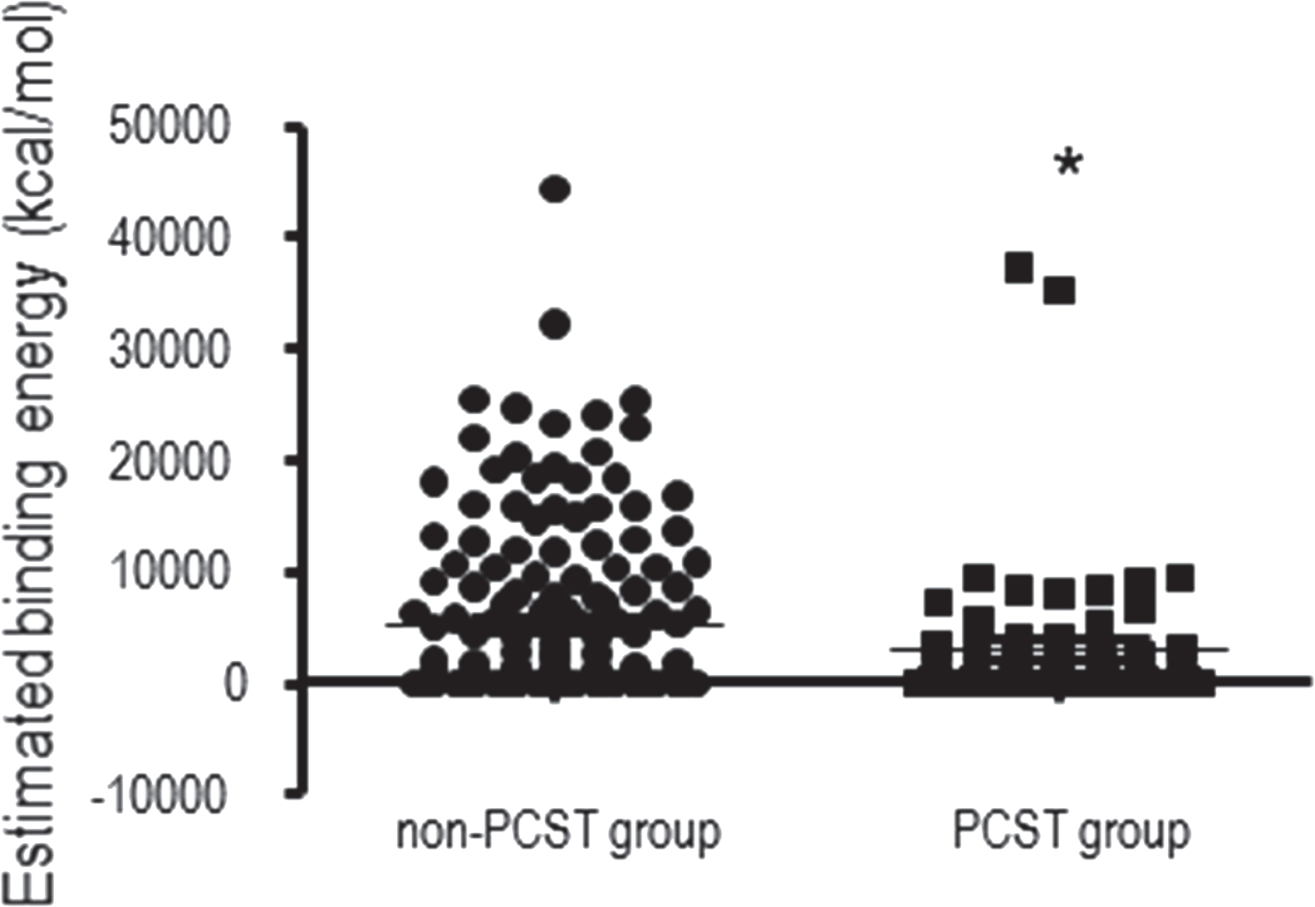
Predicted binding energies of polyP to PPK partial structural analogues with or without a Positively-Charged Semi-Tunnel. Each circle or square represented an estimated free energy of binding from the molecular docking calculations using Ap_5_ ligand and different PPK partial structural analogues.

The estimated binding energies of Ap_6_A to 233 structure chains that shared partial structural similarities to PPKs identified by the Dali program (S1 Table) were analyzed. Of the 233 structure chains, 93 had minimum estimated free energies of lower than 100 kcal/mol (37 in the range of 10~100 kcal/mol, 40 from 1~10 kcal/mol, 5 from 0~1 kcal/mol and the strongest 11 were below 0 kcal/mol), while the other 140 had minimum estimated free energies of higher than 10^2^ kcal/mol (39, 59 and 42 had 10^4^, 10^3^ and 100 kcal/mol, respectively). We divided these analogs into two groups, PCST-containing and non-PCST-containing. Analysis of the two groups of predictive minimum binding free energies identified in the PPK partial structural analogs (Fig 3) revealed that the binding energies of polyP to the PCST-containing protein group were significantly lower than those of the non-PCST-containing protein group (unpaired t-test, p<0.05). This statistical result supported the correlation between PCST and predictive polyP-binding energies.

### PCST identification in the polyP-binding subunit of PPK3

**Fig 4:**
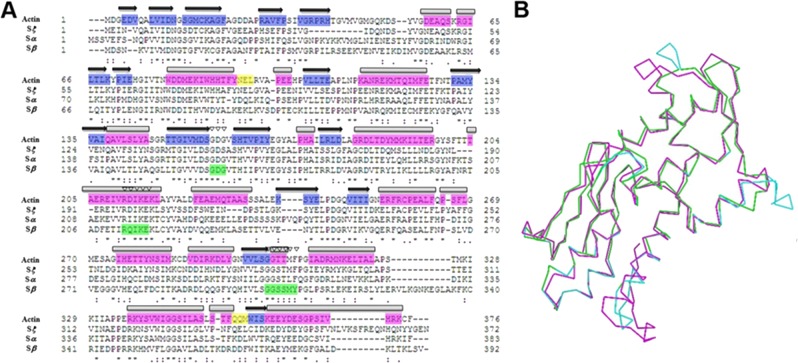
PPK3 subunits and sequences. (A) Multiple alignment of PPK3 subunit and actin amino acid sequences. Black arrows and blue highlights indicate strands; boxes and purple highlights indicate helices; yellow highlights indicate turns; inverted triangles and green highlights indicate nucleotide-binding regions. (B) The overlap of the backbone structures of the PPK3 subunits. *α*(blue); *β*(purple); *ξ*(green).

**Fig 5:**
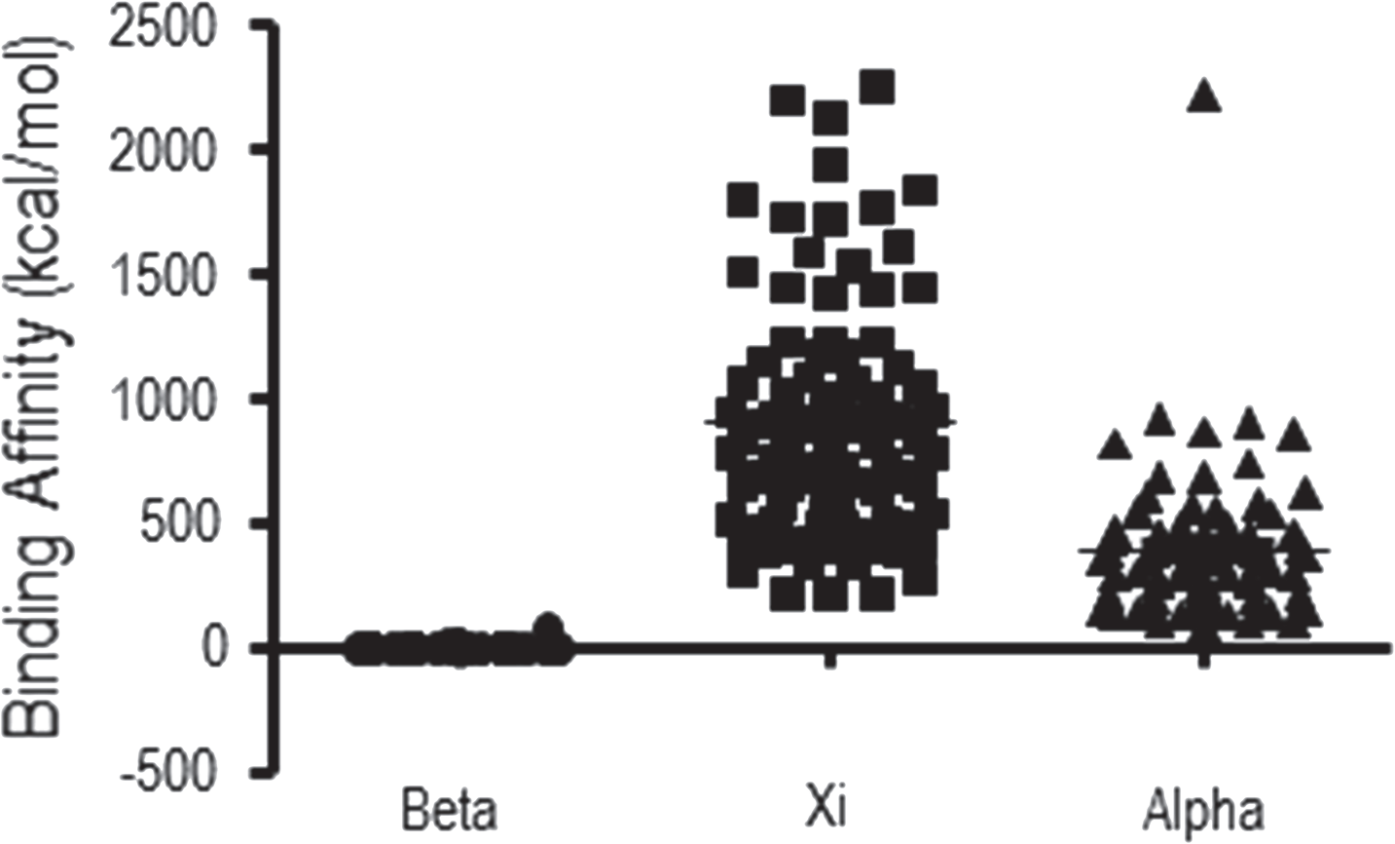
Binding affinities of polyP to subunits of PPK3. Each circle, square, or triangle represented an estimated free energy of binding from the molecular docking calculations using Ap5 ligand and different PPK3 subunit.

The structures of the three PPK3 subunits (*α*, *β* and *ξ*) were modeled based on sequence homology and multiple alignment (Fig 4A). Superimposing modeled structures of the three subunits of PPK3 revealed great structural overlap (Fig 4B). We first performed molecular docking to compare the polyP/ATP-binding capabilities of the three subunits of PPK3. The docking results illustrated that ATP could bind to all three subunits. The *α* subunit bound ATP most readily, having the lowest binding free energy value of -3.37 kcal/mol compared to -2.42 kcal/mol and -1.38 kcal/mol for *β* and *ξ*. The *β* subunit revealed high binding affinity for polyP (Fig 5). Thus, the *β* subunit could be directly involved in polyP synthesis. Positive results from docking with other short-chain polyPs (Table 2) supported this notion. Two charge calculation methods, *Gasteiger* partial [54] and PM6 semi-empirical [55], were used individually to corroborate the data, and both showed similar values for positive results (estimated minimum binding energies less than 100 kcal/mol) and negative results (estimated minimum binding energies more than 100 kcal/mol) (Table 3).

**Table 2:** Binding affinity of polyP estimated using different ligands for the PPK3 *β* subunit.

Ligand	Estimated lowest free energy of binding (kcal/mol)	Estimated highest free energy of binding (kcal/mol)	Estimated free energy of binding with highest frequency (kcal/mol)
**ATP**	**-0.91 (8%)**	**+1.37 (1%)**	**-0.48 (20%)**
**Ap4**	**+0.25 (3%)**	**+2.76 (1%)**	**+0.46 (12%)**
**Ap4A**	**+1.81 (1%)**	**+6.16 (1%)**	**+2.09 (2%)**
**Ap4U**	**+0.81 (1%)**	**+4.88 (1%)**	**+0.81 (1%)**
**Ap4-glucose**	**+3.01 (1%)**	**+6.28 (1%)**	**+3.70 (3%)**
**Ap5**	**-3.61 (3%)**	**-0.27 (1%)**	**-2.97 (5%)**
**Ap5A**	**+2.95 (2%)**	**+8.04 (1%)**	+2.95 (2%)*
**Ap5T**	**+2.92 (1%)**	**+7.91 (1%)**	**+4.36 (3%)**
**Ap6A**	**+5.06 (1%)**	**+11.68 (1%)**	+6.91 (2%)*
**Ap6T**	**+5.23 (2%)**	**+11.44 (1%)**	+5.23 (2%)*
**GTP**	**-0.78 (1%)**	**+1.18 (1%)**	-0.64 (7%)*
**Gp4**	**+0.95 (1%)**	**+4.15 (1%)**	**+2.19 (5%)**
**Gp4G**	**+0.88 (1%)**	**+5.20 (1%)**	**+1.96 (2%)**
**Gp5**	**+1.31 (3%)**	**4.36 (1%)**	**+1.96 (6%)**
**Gp5G**	**+2.58 (1%)**	**+8.25 (1%)**	+4.56 (2%)*
**ppGpp**	**+1.38 (15%)**	**+3.46 (1%)**	**+1.38 (15%)**
**pppGpp**	**+2.71 (21%)**	**+5.80 (1%)**	**+2.71 (21%)**
**Ip4**	**+1.52 (7%)**	**+4.2 (1%)**	**+1.52 (7%)**
**Ip4I**	**+0.49 (1%)**	**+5.04 (1%)**	**+1.89 (2%)**
**Ip5I**	**+3.09 (1%)**	**+8.09 (1%)**	+4.84 (2%)*

Percentages in brackets indicated estimated energy relative to total values from 100 different runs. PM6 semi-empirical charges were added to the ligand atoms. All the ligands used in the study showed positive results for the *β* subunit.

**Table 3:** Binding affinity of polyP estimated using different charge calculation methods for the PPK3 *β* subunit.

Ligand	Charge calculation method	Estimated lowest free energy of binding (kcal/mol)	Estimated highest free energy of binding (kcal/mol)	Estimated free energy of binding with highest frequency (kcal/mol)
**ATP**	***Gasteiger***	**-2.42 (4%)**	**+0.21 (1%)**	**-2.27 (7%)**
	**PM6**	**-0.91 (8%)**	**+1.37 (1%)**	**-0.48 (20%)**
**Ap5**	***Gasteiger***	**-2.40 (1%)**	**+1.23 (1%)**	**-2.17 (5%)**
	**PM6**	**-3.61 (3%)**	**-0.27 (1%)**	**-2.97 (5%)**
**Ap6A**	***Gasteiger***	**-2.71 (1%)**	**+2.27 (1%)**	**-0.92 (2%)**
	**PM6**	**+5.06 (1%)**	**+11.68 (1%)**	+6.91 (2%)*

The PM6 semi-empirical charge calculation method and *Gasteiger* partial charges showed a similar positive result for the *β* subunit. Percentages in brackets indicated estimated energy relative to total values from 100 different runs

**Fig 6:**
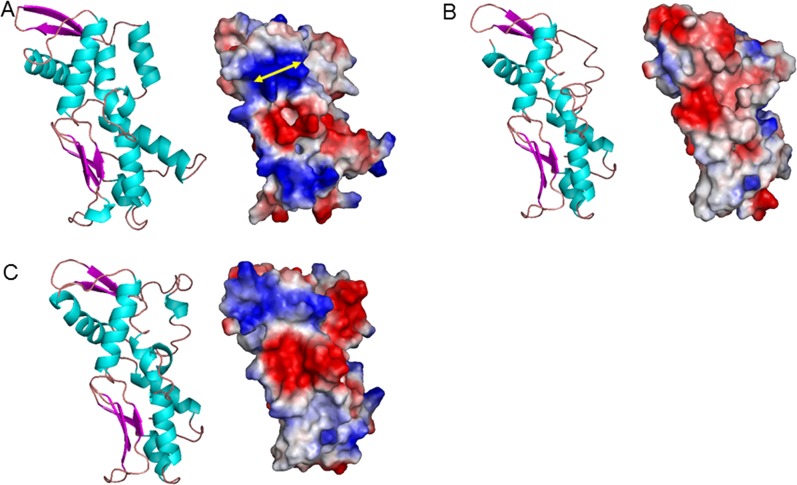
PPK3 3D structure of subunits. (A) β subunit; (B) ξ subunit; (C) α subunit. Protein structure (left) and protein surface graphic (right). Colors on graphics represent surface charges of the 3D structure of the protein. Blue = positive; Red = negative. Yellow double end arrows indicate observed strip of PCST.

We then determined the presence of the PCST structure in PPK3. Surface electrostatics analyses showed more widespread distributions of basic residues in the *α* and *β* subunits compared to the *ξ* subunit (Fig 6), probably facilitating the interactions of these subunits with the phosphate of a nucleotide/polyP. Furthermore, the *β* subunit possessed a typical PCST structure. Combined with the docking results and the PCST structure identification in all the PPKs, our data suggested the capability of PPK3*β* to bind polyP. Utilization of molecular docking calculations revealed high nucleotide affinity of the three PPK3 subunits, but only the *β* subunit showed specific polyP affinity. Because a PCST existed in the *β* subunit, but not in the *α* or *ξ* subunits, our data supported the high correlation of the PCST to polyP functionality.

## Discussion

Mammalian polyP plays important regulatory roles through its interactions with related proteins [56–61]. The identification of polyP-synthesizing enzymes in higher eukaryotes remains unresolved, and the polyP-binding sequence and structural features are largely unknown [62]. We observed a PCST structure in PPKs, the enzymes that perform polyP synthetic reactions, and revealed the secondary structure compositional diversity of the PCSTs. The local semi-closure of the tunnel locks positively-charged metal ions (forming bonds with polyP) in place, and the charged polar (acidic / basic) amino acids located inside the tunnel interact with phosphates of the polyP chain through water hydrolysis. The previously described structure of the polyP-dependent hexokinase (HK) family showed flexible subdomain structures that enabled the orthologous gene products in higher organisms to lose the ability to utilize polyP. We deduced that the polyP binding affinities varied among the genetic variations of the PCST structures from different homologs. This indicated possible future directions for the identification of potential subgroups in the PCST-containing proteins based on the binding pocket shape and more accurate structural classification methods. The mechanisms in different polyP-related proteins need to be investigated further.

To discover more polyP-related genes, and considering that structural information is more evolutionarily conserved than primary sequence [63], we identified partial structural analogs (proteins possessing partial structural similarity) of PPKs (see Supporting Information). We envisioned that these proteins would have polyP-binding potential and a PCST structure, and thus their homologous proteins would retain polyP-binding characteristics in higher organisms. Our results confirmed a substantial number of PCST-containing proteins among the partial structural analogs of PPKs (62 in 242) based on the minimum binding energy estimated. Some widely distributed positive charges on protein surfaces may generate binding energies that are unspecific to polyP-binding capacities. For example, PPK4 possesses more positive charges than the other PPKs, as shown in Fig 1, while displaying much more diverse values in the molecular docking results of Fig 2. Furthermore, the molecular docking results showed that PCST-containing proteins bind polyP more readily than non-PCST-containing proteins. We therefore concluded that the PCST is probably a characteristic feature of polyP-binding structures, and its presence can be potentially utilized to identify polyP-related proteins. The PCST structure is probably of biochemical significance for polyP functions in cellular processes. The functions of the PCST highlight the potential regulatory mechanisms through which PCST-containing proteins can be dynamically affected by the polyP in cells and the microenvironment [64]. Additionally, with the recent advances in systems biology and protein engineering, engineered proteins that are modified by adding a PCST may allow for manipulation of polyP as an energy and phosphate source.

PPK3 enzymatic activity and sequences were first identified in *Dictyostelium discoideum* [42]. We simulated the structures of three PPK3 subunits (*α*, *β* and *ξ*) based on the reported homology. Molecular docking supported the finding that the subunits of PPK3 from the actin family possessed a nucleotide-binding function. Meanwhile, the PCST found in the modeled *β* subunit of PPK3 generated a theoretical polyP-binding site. PPK3 was the only eukaryotic PPK that had homology to human proteins [42]. Our study provided useful information for the identification of human PCST-containing proteins and predicted that the PCST was a structure that could bind polyP. We also noticed that 15 of the 56 proteins with minimum estimated free energy of less than 10 kcal/mol were mitochondrial proteins. This result suggested the involvement of polyP in energy metabolism and mitochondrial functions. Recent reports have shown that polyP synthesis was dependent on the energy metabolic state of mitochondria [18,65]. We concluded that a set of mitochondrial proteins were probably involved in polyP synthesis and utilization.

We believe that in-depth research on PCST-containing proteins, which are potential polyP-binding proteins, will shed light on what physiological roles polyP plays, and how polyP is involved in human diseases. The identification of PCSTs in different proteins will allow the discovery of new pathways through which polyP is utilized and provide a new understanding of how phosphate reserves are utilized.

## Conclusions

In this study, we used bioinformatics approaches to search for polyP-related proteins. Subsequently we observed a structure on the surface of proteins that is a potential binding site for polyP. This structure will allow the discovery of more polyP-related proteins and help reveal the phosphate metabolism and regulatory functions of polyP in higher organisms.

## Supporting Information

S1 Table List of partial structural analogs and their characteristics. Human homologous genes of partial structural analogs are listed in the Table. The parameters calculated using the Dali program [Z-score, RMSD, length of the alignment (LALI), number of aligned residues (NRES) and Identity (%)] are also included. Docking calculations were performed using the Docking Server. The MMFF94 force field was used for energy minimization and *Gasteiger* partial charges were added to Ap_6_A ligand atoms. Each docking experiment was derived from 10 different runs that were set to terminate after a maximum of 250,000 energy evaluations. The estimated minimum energy, indicative of polyP-binding potential, was added to the Table. The unidentified results originated from errors in the surface electrostatics analysis or docking calculations. Attempts were also made to determine the presence of PCST structures in these partial structural analogs. PC, with strong positive charges; ST, semi-tunnels without strong positive charges; unidentified, no obvious characteristics. Asterisks (*) beside the UniProt accession numbers indicated the E-value of sequence matches to polyP-related motifs. **E-value < 0. 01. *E-value <0. 1 and >0. 01.
(PDF)

S1 Text Identification of partial structural analogs of PPKs. (PDF)
