# Supplementary material for: Positively-Charged Semi-Tunnel Is a Structural and Surface Characteristic of Polyphosphate-Binding Proteins: An In-Silico Study
Source: PLoS One. 2015 Apr 16;10(4):e0123713. doi: 10.1371/journal.pone.0123713 (PMC4400040; doi:10.1371/journal.pone.0123713)
Supplement: S1 Table — Human homologous genes of partial structural analogs are listed in the Table. The parameters calculated using the Dali program [Z-score, RMSD, length of the alignment (LALI), number of aligned residues (NRES) and Identity (%)] are also included. Docking calculations were performed using the Docking Server. The MMFF94 force field was used for energy minimization and Gasteiger partial charges were added to Ap6A ligand atoms. Each docking experiment was derived from 10 different runs that were set to terminate after a maximum of 250,000 energy evaluations. The estimated minimum energy, indicative of polyP-binding potential, was added to the Table. The unidentified results originated from errors in the surface electrostatics analysis or docking calculations. Attempts were also made to determine the presence of PCST structures in these partial structural analogs. PC, with strong positive charges; ST, semi-tunnels without strong positive charges; unidentified, no obvious characteristics. Asterisks (*) beside the UniProt accession numbers indicated the E-value of sequence matches to polyP-related motifs. **E-value < 0. 01. *E-value <0. 1 and >0. 01. (PDF) [file pone.0123713.s001.pdf]

	Human Homologs and UniProt Accession	Structural Analogs and UniProt Accession	PDB Chain	Estimated PolyP Binding Energy （kcal/ mol）	Identification of PCST	Source	Cell Type	Z-Score	RMSD	LALI	NRES	Identity (%)	Protein Name
**PPK1**	ACADVL/P49748	ACADVL/P49748	2uxw-A	Unidentified	ST	*Homo sapiens*	HE；M	2.2	14.9	133	567	3	Very long-chain specific acyl-CoA dehydrogenase,mitochondrial
**PPK1**	ACE/P12821	DCP/P24171	1y79-1	+153.60	ST,PC	*Escherichia coli*	P；B	5	16.7	178	680	4	Peptidyl-dipeptidase
**PPK1**	ACE2/Q9BYF1	ACE2/Q9BYF1	1r4l-A	+147.01	ST	*Homo sapiens*	HE；M	3.3	15.7	152	597	6	Angiotensin-converting enzyme 2
**PPK1**	ACTN1/P12814	ABPA/P05095*	1g8x-A	+3.32	ST,PC	*Dictyostelium discoideum*	LE	6.2	31.6	146	1009	5	Alpha-actinin A
**PPK1**	ACTN2/P35609	ACTN2/P35609	1hci-A	+5.68	Unidentified	*Homo sapiens*	HE；M	5.8	71.5	202	475	9	Alpha-actinin-2
**PPK1**	ADCY1/Q08828,ADCY8/P40145	CYA/P40136	1sk6-A	+2.38e+03	ST,PC	*Bacillus anthracis*	P；B	2.9	4.4	81	481	9	Calmodulin-sensitive adenylate cyclase
**PPK1**	AHSP/Q9NZD4	AHSP/Q9NZD4	1w0b-A	+798.34	ST	*Homo sapiens*	HE；M	5	5	90	102	8	Alpha-hemoglobin-stabilizing protein
**PPK1**	AIDA/Q96BJ3*	AIDA/Q8C4Q6*	1ug7-A	+2.02e+04	ST	*Mus musculus*	HE；M	3.1	4.8	82	128	15	Axin interactor,dorsalization-associated protein
**PPK1**	AMPH/P49418	AMPH/Q7KLE5	1uru-A	+564.74	Unidentified	*Drosophila melanogaster*	HE	3.2	20.3	107	217	10	Amphiphysin
**PPK1**	ANAPC7/Q9UJX3*	ANAPC7/Q9UJX3*	3ffl-A	+533.28	ST,PC	*Homo sapiens*	HE；M	2.2	11.1	82	126	9	Anaphase-promoting complex subunit 7
**PPK1**	APOE/P02649	APOE/P02649	1b68-A	+1.23e+04	ST,PC	*Homo sapiens*	HE；M	5.7	4.6	97	138	11	Apolipoprotein E
**PPK1**	APP/P05067**	APP/P05067**	1rw6-A	+152.80	ST,PC	*Homo sapiens*	HE；M	6.3	4.4	98	192	7	Amyloid beta A4 protein
**PPK1**	ARFIP2/P53365	ARFIP2/P53365	1i4l-A	+143.57	ST	*Homo sapiens*	HE；M	2.6	18	109	185	5	ADP-ribosylation factor-interacting protein 2
**PPK1**	ARHGEF3/Q9NR81	ARHGEF3/Q91X46	2z0q-A	+3.52	PCST	*Mus musculus*	HE；M	2.9	6.7	125	321	11	Rho guanine nucleotide exchange factor 3
**PPK1**	ATP5G1/P05496,ATP5G2/Q06055, ATP5G3/P48201	ATPE/P68699	1l6t-A	+4.75	ST	*Escherichia coli*	P；B	4.2	4.7	74	79	4	ATP synthase subunit c
**PPK1**	BAG1/Q99933*	BAG1/Q99933*	1hx1-B	+85.20	ST	*Homo sapiens*	HE；M	4.2	3.7	72	112	10	BAG family molecular chaperone regulator 1
**PPK1**	BAG4/O95429	BAG4/O95429	1m62-A	+1.37e+03	ST,PC	*Homo sapiens*	HE；M	5.2	3.6	71	87	7	BAG family molecular chaperone regulator 4
**PPK1**	BAG5/Q9UL15	BAG5/Q9UL15	2d9d-A	+1.03e+04	PC	*Homo sapiens*	HE；M	4.7	5.5	85	89	7	BAG family molecular chaperone regulator 5
**PPK1**	BAIAP2/Q9UQB8	BAIAP2/Q9UQB8	1y2o-A	+6.55	Unidentified	*Homo sapiens*	HE；M	2.9	18.5	110	248	9	Brain-specific angiogenesis inhibitor 1-associated protein 2
**PPK1**	BCKDK/O14874	BCKDK/Q00972	1gkx-A	+45.93	ST,PC	*Rattus norvegicus*	HE；M	3.1	5.9	90	308	8	[3-methyl-2-oxobutanoate dehydrogenase [lipoamide]] kinase,mitochondrial
**PPK1**	BUB1/O43683	BUB1/P41695	3esl-A	+7.61e+03	ST,PC	*Saccharomyces cerevisiae*	LE	2.4	10.1	84	195	10	Mitotic checkpoint serine/threonine-protein kinase BUB1
**PPK1**	CAB39/Q9Y376	CAB39/Q9Y376	3gni-A	+39.58	ST,PC	*Homo sapiens*	HE；M	4.1	5.8	88	335	7	Calcium-binding protein 39
**PPK1**	CBL/P22681	CBL/P22681	1b47-A	+278.84	ST,PC	*Homo sapiens*	HE；M	3.4	17.4	133	304	9	E3 ubiquitin-protein ligase CBL
**PPK1**	CHRNA1/P02708	CHRNA1/P02711	1oed-A	+179.03	ST	*Torpedo marmorata*	HE	7.5	3.8	90	127	7	Acetylcholine receptor subunit alpha
**PPK1**	CHRNB1/P11230	CHRNB1/P02712**	1oed-B	+435.36	PCST	*Torpedo marmorata*	HE	6.3	3.6	90	127	9	Acetylcholine receptor subunit beta
**PPK1**	CHRND/Q07001	CHNRD/P02718	1oed-C	+45.83	ST	*Torpedo marmorata*	HE	6.5	2.8	89	127	9	Acetylcholine receptor subunit delta
**PPK1**	CHRNG/P07510	CHRNG/P02714	1oed-E	+91.88	PCST	*Torpedo marmorata*	HE	7.6	3.5	90	128	8	Acetylcholine receptor subunit gamma
**PPK1**	CLINT1/Q14677	CLINT1/Q14677	2v8s-V	+8.61e+03	ST,PC	*Homo sapiens*	HE；M	6	2.6	73	93	10	Clathrin interactor 1
**PPK1**	CYCS/P99999	CYCA/P00149	1a7v-A	+3.17e+03	PCST	*Rhodopseudomonas palustris*	P；B	2.8	5.3	82	125	6	Cytochrome c

**PPK1**	DAAM1/Q9Y4D1	DAAM1/Q9Y4D1	2j1d-G	+2.21	ST	*Homo sapiens*	HE； M	4.7	14	142	394	3	Disheveled-associated activator of morphogenesis 1
**PPK1**	DIAPH1/O60610	DIAPH1/O08808	1v9d-A	+1.73	ST,PC	*Mus musculus*	HE； M	6.2	13	137	308	7	Protein diaphanous homolog 1
**PPK1**	DOCK9/Q9BZ29*	DOCK9/Q9BZ29*	2wm9-A	+2.36	PCST	*Homo sapiens*	HE； M	4.1	4.6	91	407	5	Dedicator of cytokinesis protein 9
**PPK1**	EXOC3/O60645*	SEC6/P32844*	2fji-1	+2.35	ST	*Saccharomyces cerevisiae*	LE	5.5	17.1	165	399	7	Exocyst complex component 3
**PPK1**	FKBP4/Q02790*	FKBP35/Q8I4V8*	2fbn-A	+657.36	ST,PC	*Plasmodium falciparum*	LE	5.6	9.4	100	153	4	Peptidyl-prolyl cis-trans isomerase FKBP4
**PPK1**	FKBP5/Q13451	FKBP5/Q9XSH5*	1kt1-A	-0.43	PCST	*Saimiri boliviensis*	HE； M	4.8	11.6	83	374	12	Peptidyl-prolyl cis-trans isomerase FKBP5
**PPK1**	FTCD/O95954	FTCD/O88618	2pfd-A	+1.89	PCST	*Rattus norvegicus*	HE； M	4	9.3	99	540	6	Formimidoyltransferase-cyclodeaminase
**PPK1**	FTH1/P02794	FTH1/P02794	2chi-A	+6.57e+03	ST,PC	*Homo sapiens*	HE； M	3.1	5.5	89	172	10	Ferritin heavy chain
**PPK1**	FTL/P02792	FTL/P02792	2fg8-A	+1.60e+04	ST	*Homo sapiens*	HE； M	3	7.3	94	174	9	Ferritin light chain
**PPK1**	GARS/P41250	GLYQ/Q9WY59	1j5w-A	+106.52	ST,PC	*Thermotoga maritima*	P； B	5.6	2.9	73	276	11	Glycyl-tRNA synthetase
**PPK1**	GBP1/P32455	GBP1/P32455	1dg3-A	-0.08	ST,PC	*Homo sapiens*	HE； M	5.7	13.5	119	540	4	Interferon-induced guanylate-binding protein 1
**PPK1**	GCDH/Q92947	GCDH/Q92947	2r0n-A	+5.48e+03	ST	*Homo sapiens*	HE； M	2.4	9.1	91	390	2	Glutaryl-CoA dehydrogenase,mitochondrial
**PPK1**	GGA1/Q9UJY5	GGA1/Q9UJY5	1naf-A	+6.48	ST,PC	*Homo sapiens*	HE； M	6.1	4.9	83	124	7	ADP-ribosylation factor-binding protein GGA1
**PPK1**	GGA3/Q9NZ52	GGA3/Q9NZ52	1wr6-A	+6.12e+03	ST	*Homo sapiens*	HE； M	5.9	3.2	80	90	10	ADP-ribosylation factor-binding protein GGA3
**PPK1**	GINS4/Q9BRT9	GINS4/Q9BRT9	2eho-A	+244.76	ST,PC	*Homo sapiens*	HE； M	3	14.2	95	161	8	DNA replication complex GINS protein SLD5
**PPK1**	GIT1/Q9Y2X7	GIT1/Q9Z272	2jx0-A	+9.26e+03	PC	*Rattus norvegicus*	HE； M	2.3	4.2	77	131	4	ARF GTPase-activating protein GIT1
**PPK1**	GJB2/P29033	GJB2/P29033	2zw3-A	+3.20e+03	PCST	*Homo sapiens*	HE； M	5.3	3.3	95	201	8	Gap junction beta-2 protein
**PPK1**	GSTA1/P08263	GSTA1/P00502	1ev9-D	+2.26e+03	PCST	*Rattus norvegicus*	HE； M	4.7	3.8	81	216	2	Glutathione S-transferase A1
**PPK1**	GSTA4/O15217	GSTA4/P24472*	1guk-A	+5.33e+03	ST,PC	*Mus musculus*	HE； M	4.7	3.6	80	215	9	Glutathione S-transferase A4
**PPK1**	GSTM1/P09488*	GSTM1/P04905	3fyg-A	+6.56e+03	PCST	*Rattus norvegicus*	HE； M	3	5.6	74	217	5	Glutathione S-transferase Mu 1
**PPK1**	GSTP1/P09211	GST2/P46427	1tu8-A	+2.51e+03	PCST	*Onchocerca volvulus*	HE	3.4	3.6	75	208	11	Glutathione S-transferase P
**PPK1**	GSTT2B/P0CG30*	GSTT2B/P0CG30*	3ljr-A	+386.20	PCST	*Homo sapiens*	HE； M	3.7	6.6	91	244	8	Glutathione S-transferase theta-2B
**PPK1**	GSTZ1/O43708	GSTZ1/O43708	1fw1-A	+2.88e+03	PCST	*Homo sapiens*	HE； M	3.1	3.6	75	208	3	Maleylacetoacetate isomerase
**PPK1**	HIP1R/O75146	HIP1R/O75146	1r0d-A	+5.34e+03	ST,PC	*Homo sapiens*	HE； M	4.8	8.4	118	194	8	Huntingtin-interacting protein 1-related protein
**PPK1**	HPGDS/O60760*	GST-5/Q09596*	1zl9-A	+81.19	PCST	*Caenorhabditis elegans*	HE	4.7	6.2	82	207	5	Hematopoietic prostaglandin D synthase
**PPK1**	HSCB/Q8IWL3	HSCB/Q8IWL3	3bvo-A	+1.31	PCST	*Homo sapiens*	HE； M	4.5	3.7	78	197	3	Iron-sulfur cluster co-chaperone protein HscB,mitochondrial
**PPK1**	HSD11B1/P28845	HSD11B1/P28845	3ch6-B	+1.59e+04	ST,PC	*Homo sapiens*	HE； M	2.2	3.8	77	279	12	Corticosteroid 11-beta-dehydrogenase isozyme 1
**PPK1**	HSPA8/P11142	HSP-1/P09446	2p32-A	+128.72	Unidentified	*Caenorhabditis elegans*	HE	3.4	8.1	70	82	9	Heat shock cognate 71 kDa protein
**PPK1**	IL12A/P29459	IL12A/P29459	1f45-B	+3.68e+03	ST,PC	*Homo sapiens*	HE； M	4	4.3	75	133	7	Interleukin-12 subunit alpha
**PPK1**	KLC2/Q9H0B6*	KLC2/Q9H0B6*	3ceq-B	-0.43	ST,PC	*Homo sapiens*	HE； M	3.3	16.2	90	269	7	Kinesin light chain 2
**PPK1**	LARS/Q9P2J5,LARS2/Q15031	LEUS/O58698*	1wz2-A	+3.53	ST,PC	*Pyrococcus horikoshii*	P； A	4.3	9.9	119	948	10	Probable leucyl-tRNA synthetase
**PPK1**	LRPAP1/P30533*	LRPAP1/P30533*	2ftu-A	+2.80e+03	ST	*Homo sapiens*	HE； M	4.5	6.8	89	118	12	Alpha-2-macroglobulin receptor-associated protein
**PPK1**	MARS/P56192,MARS2/Q96GW9	METG/P00959	1pfv-A	+4.14e+03	PCST	*Escherichia coli*	P； B	3.9	5.6	100	547	12	Methionyl-tRNA synthetase

**PPK1**	MET/P08581*	MET/P08581*	3bux-B	+5.44e+03	PCST	*Homo sapiens*	HE; M	3	19.3	120	305	8	Hepatocyte growth factor receptor
**PPK1**	MMAB/Q96EY8	MMAB/Q96EY8	2idx-C	+1.93e+04	ST,PC	*Homo sapiens*	HE; M	2.7	10.8	95	180	9	Cob(I)yrinic acid a,c-diamide adenosyltransferase,mitochondrial
**PPK1**	MRRF/Q96E11	FRR/Q9X1B9	1dd5-A	+3.04	ST,PC	*Thermotoga maritima*	P; B	7.4	3.5	93	184	6	Ribosome-recycling factor,mitochondrial
**PPK1**	MT-CO3/P00414	CTAE/P06030	1qle-C	+1.46	ST	*Paracoccus denitrificans*	P; B	4.9	3.4	96	273	7	Cytochrome c oxidase subunit 3
**PPK1**	MT-CYB/P00156	PETB/Q00471	1q90-B	+3.98e+03	PCST	*Chlamydomonas reinhardtii*	HE; P	3.8	5.6	95	212	4	Cytochrome b
**PPK1**	NCBP1/Q09161	NCBP1/Q09161	1h6k-C	-0.20	PCST	*Homo sapiens*	HE; M	2.9	3.4	84	733	6	Nuclear cap-binding protein subunit 1
**PPK1**	NLN/Q9BYT8	NLN/P42676	1i1i-P	+3.99	PCST	*Rattus norvegicus*	HE; M	6.3	17.1	186	665	6	Neurolysin,mitochondrial
**PPK1**	NSMCE2/Q96MF7*	MMS21/P38632*	3htk-C	+5.00	ST,PC	*Saccharomyces cerevisiae*	LE	4.1	3.8	81	254	6	E3 SUMO-protein ligase NSE2
**PPK1**	NUP93/Q8N1F7*	NIC96/P34077**	2qx5-A	+5.33	PCST	*Saccharomyces cerevisiae*	LE	4.6	12.1	112	569	6	Nuclear pore complex protein Nup93
**PPK1**	OXSM/Q9NWU1*	FAS2/P19097*	3hmj-A	+6.05	PCST	*Saccharomyces cerevisiae*	LE	3.1	5.8	68	1750	4	3-oxoacyl-[acyl-carrier-protein] synthase,mitochondrial
**PPK1**	P4HA1/P13674*	P4HA1/P13674*	2v5f-A	+7.77e+03	Unidentified	*Homo sapiens*	HE; M	2.4	3.5	60	97	8	Prolyl 4-hydroxylase subunit alpha-1
**PPK1**	PDCD10/Q9BUL8	PDCD10/Q9BUL8	3l8i-B	+0.29	PCST	*Homo sapiens*	HE; M	2.7	3.7	76	201	9	Programmed cell death protein 10
**PPK1**	PDCD6IP/Q8WUM4	PDCD6IP/Q8WUM4	2oex-A	+2.70	ST	*Homo sapiens*	HE; M	5.3	7.6	100	342	9	Programmed cell death 6-interacting protein
**PPK1**	PDE1B/Q01064	PDE1B/Q01064	1taz-A	+1.49e+04	ST,PC	*Homo sapiens*	HE; M	4.2	3.8	79	322	6	Calcium/calmodulin-dependent 3',5'-cyclic nucleotide phosphodiesterase 1B
**PPK1**	PDK4/Q16654*	PDK4/Q16654*	2e0a-A	+73.30	ST,PC	*Homo sapiens*	HE; M	4.1	4.4	119	358	5	[Pyruvate dehydrogenase [lipoamide]] kinase isozyme 4,mitochondrial
**PPK1**	PICALM/Q13492*	PICALM/O55012*	1hf8-A	+1.84e+04	ST,PC	*Rattus norvegicus*	HE; M	5.4	3.9	92	263	5	Phosphatidylinositol-binding clathrin assembly protein
**PPK1**	PPID/Q08752	PPID/P26882	1ihg-A	-0.75	PCST	*Bos taurus*	HE; M	5.2	7.6	91	364	8	Peptidyl-prolyl cis-trans isomerase D
**PPK1**	PSIP1/O75475	PSIP1/O75475	3f9k-a	+1.06e+04	ST,PC	*Homo sapiens*	HE; M	4.6	4.3	67	94	7	PC4 and SFRS1-interacting protein
**PPK1**	PSME1/Q06323	PSME1/Q06323	1avo-B	+5.64	ST	*Homo sapiens*	HE; M	4.7	9.5	102	140	4	Proteasome activator complex subunit 1
**PPK1**	PTDSS1/P48651,PTDSS2/Q9BVG9	PSSA/P44704**	3hsi-A	+7.95e+03	PCST	*Haemophilus influenzae*	P; B	21.1	3.3	289	447	12	Phosphatidylserine synthase
**PPK1**	PTK2/Q05397	PTK2/Q00944	1qvx-A	+5.37e+03	PCST	*Gallus gallus*	HE	3.4	9.4	92	134	10	Focal adhesion kinase 1
**PPK1**	RAC1/P63000	RAC1/P63000	2nz8-B	+4.68	ST,PC	*Homo sapiens*	HE; M	2.9	7.7	127	279	9	Ras-related C3 botulinum toxin substrate 1
**PPK1**	RAMP1/O60894	RAMP1/O60894	2yx8-A	+1.63e+03	ST	*Homo sapiens*	HE; M	4.5	3.7	68	81	9	Receptor activity-modifying protein 1
**PPK1**	RAPGEF4/Q8WZA2	RAPGEF4/Q9EQZ6*	3cf6-E	+1.57e+04	ST,PC	*Mus musculus*	HE; M	5.7	18.5	147	627	5	Rap guanine nucleotide exchange factor 4
**PPK1**	RTN4R/Q9BZR6	NGR/P30820	1yux-B	+2.33e+04	ST,PC	*Desulfovibrio vulgaris*	P; B	3.1	7	99	202	6	Reticulon-4 receptor
**PPK1**	SARS/P49591,SARS2/Q9NP81*	SERS/P34945	1set-A	+248.07	ST,PC	*Thermus thermophilus*	P; B	2.9	24.8	129	421	5	Seryl-tRNA synthetase
**PPK1**	SFN/P31947	SFN/P31947	1ywt-A	+5.62	ST,PC	*Homo sapiens*	HE; M	4.1	12.2	101	223	10	14-3-3 protein sigma
**PPK1**	SOS1/Q07889	SOS1/Q07889	1nvx-S	+2.62e+03	ST,PC	*Homo sapiens*	HE; M	3.4	20.2	153	448	6	Son of sevenless homolog 1
**PPK1**	SPAST/Q9UBP0	SPAST/Q9UBP0	3eab-A	+42.18	PCST	*Homo sapiens*	HE; M	5.5	3.5	74	89	7	Spastin
**PPK1**	SPTA1/P02549	SPTA1/P02549	1owa-A	+3.48	ST,PC	*Homo sapiens*	HE; M	7.6	2.6	88	156	6	Spectrin alpha chain,erythrocyte
**PPK1**	SPTAN1/Q13813	SPTAN1/P07751	1aj3-A	+443.27	ST,PC	*Gallus gallus*	HE	6.9	2.7	83	98	5	Spectrin alpha chain,brain
**PPK1**	SPTB/P11277	SPTB/P11277	1s35-A	+14.61	ST	*Homo sapiens*	HE; M	7.2	17.7	144	211	8	Spectrin beta chain,erythrocyte
**PPK1**	SPTBN1/Q01082	SPTBN1/Q01082	3edv-A	Unidentified	ST	*Homo sapiens*	HE; M	5	13.5	112	322	7	Spectrin beta chain,brain 1

**PPK1**	STAT1/P42224	STAT1/P42224	1bf5-A	+60.37	ST,PC	*Homo sapiens*	HE； M	6	22	187	545	7	Signal transducer and activator of transcription 1-alpha/beta
**PPK1**	STAT3/P40763	STAT3/P42227	1bg1-A	+21.77	ST,PC	*Mus musculus*	HE； M	6.3	23	197	559	7	Signal transducer and activator of transcription 3
**PPK1**	STAT5A/P42229	STAT5A/P42230	1y1u-A	+26.42	ST,PC	*Mus musculus*	HE； M	7	24.1	190	544	6	Signal transducer and activator of transcription 5A
**PPK1**	STIP1/P31948*	STIP1/P31948*	1elw-A	+36.15	PCST	*Homo sapiens*	HE； M	2.3	9.4	68	117	3	Stress-induced-phosphoprotein 1
**PPK1**	STX12/Q86Y82	STX12/Q86Y82	2dnx-A	+696.55	ST,PC	*Homo sapiens*	HE； M	6.3	3.1	98	130	7	Syntaxin-12
**PPK1**	STX1A/Q16623	STX1A/P32851	1br0-A	+99.28	ST	*Rattus norvegicus*	HE； M	6.3	5.2	97	120	8	Syntaxin-1A
**PPK1**	TBCA/O75347	RBL2/P48606	1qsd-A	+47.27	Unidentified	*Saccharomyces cerevisiae*	LE	5	3	76	102	8	Tubulin-specific chaperone A
**PPK1**	TCEA3/O75764	TCEA3/P23881	1wjt-A	+142.61	ST,PC	*Mus musculus*	HE； M	2.4	13.9	70	103	7	Transcription elongation factor A protein 3
**PPK1**	TDP1/Q9NUW8*	TDP1/Q9NUW8*	1jy1-A	+3.22e+04	ST,PC	*Homo sapiens*	HE； M	15.8	4	254	439	14	Tyrosyl-DNA phosphodiesterase 1
**PPK1**	TERT/O14746	TERT/O77448	2r4g-A	+2.13e+03	PCST	*Tetrahymena thermophila*	LE	4.4	4.9	89	250	7	Telomerase reverse transcriptase
**PPK1**	THOP1/P52888	THOP1/P52888	2o36-A	+1.99	ST,PC	*Homo sapiens*	HE； M	6.5	17.1	186	654	8	Thimet oligopeptidase
**PPK1**	TLN1/Q9Y490	TLN1/P26039	2b0h-A	+1.32e+04	Unidentified	*Mus musculus*	HE； M	4.8	3.9	85	137	9	Talin-1
**PPK1**	TLN2/Q9Y4G6	RHEA/Q9VSL8	3fyq-A	+1.83e+04	ST,PC	*Drosophila melanogaster*	HE	4.8	13.5	95	179	3	Talin-2
**PPK1**	TOM1/O60784*	TOM1/O60784*	1wrd-A	+632.33	ST	*Homo sapiens*	HE； M	6	4.2	86	98	14	Target of Myb protein 1
**PPK1**	TOP1/P11387	TOP1/P11387	1sc7-A	-1.93	PCST	*Homo sapiens*	HE； M	4.6	9	97	567	6	DNA topoisomerase 1
**PPK1**	TRIP10/Q15642	TRIP10/Q15642	2ke4-A	+3.18	ST,PC	*Homo sapiens*	HE； M	4.3	3	80	98	8	Cdc42-interacting protein 4
**PPK1**	TSG101/Q99816	STP22/P25604	2f66-A	+19.74	ST	*Saccharomyces cerevisiae*	LE	4.1	3.2	64	65	6	Tumor susceptibility gene 101 protein
**PPK1**	TSN/Q15631	TRSN/Q7JVK6	2qrx-A	+5.84e+03	ST	*Drosophila melanogaster*	HE	4.6	4	89	185	7	Translin
**PPK1**	UBE4B/O95155	UFD2/P54860	3m62-A	Unidentified	ST,PC	*Saccharomyces cerevisiae*	LE	2.2	5.4	102	955	9	Ubiquitin conjugation factor E4 B
**PPK1**	USP8/P40818*	USP8/P40818*	2a9u-A	-2.27	ST,PC	*Homo sapiens*	HE； M	5.9	11.8	99	134	8	Ubiquitin carboxyl-terminal hydrolase 8
**PPK1**	VAV1/P15498	VAV1/P15498	3bji-A	+10.55	ST,PC	*Homo sapiens*	HE； M	2.6	11.2	140	372	8	Proto-oncogene vav
**PPK1**	VCL/P18206*	VCL/P12003*	1qkr-B	+439.53	ST,PC	*Gallus gallus*	HE	2.9	7.5	93	180	9	Vinculin (Metavinculin)
**PPK1**	VPS37A/Q8NEZ2,VPS37B/Q9H9H, VPS37C/A5D8V6,VPS37D/Q86XT2	SRN2/Q99176	2f66-F	Unidentified	PC	*Saccharomyces cerevisiae*	LE	4	2.7	63	63	13	Vacuolar protein sorting-associated protein
**PPK1**	VPS4A/Q9UN37	VPS4A/Q9UN37	1yxr-A	+946.16	ST	*Homo sapiens*	HE； M	4.9	3	72	77	13	Vacuolar protein sorting-associated protein 4A
**PPK1**	VTI1B/Q9UEU0*	VTI1B/O88384*	2qyw-A	+6.54e+03	ST	*Mus musculus*	HE； M	6.6	4.1	80	96	9	Vesicle transport through interaction with t-SNAREs homolog 1B
**PPK1**	YWHAZ/P63104	YWHAZ/P63104	1qja-A	+8.92	PCST	*Homo sapiens*	HE； M	2.4	5.4	89	217	12	14-3-3 protein zeta/delta
**PPK2**	ADK/P55263	ADK/P69441	1ank-A	+323.45	ST	*Escherichia coli*	P； B	9.1	3.6	157	214	11	Adenosine kinase
**PPK2**	AK1/P00568	AK1/P00568	1z83-A	+200.83	PCST	*Homo sapiens*	HE； M	10.2	3.6	161	195	14	Adenylate kinase isoenzyme 1
**PPK2**	AK2/P54819*	AK2/P54819*	2c9y-A	+3.32	PCST	*Homo sapiens*	HE； M	10.9	3.2	164	218	13	Adenylate kinase 2,mitochondrial
**PPK2**	AK3/Q9UIJ7*	AK3/P08760*	2ak3-A	-3.96	PCST	*Bos taurus*	HE； M	10.2	3.6	162	226	10	GTP:AMP phosphotransferase,mitochondrial
**PPK2**	AK4/P27144*	AK4/P27144*	2bbw-A	+4.96	PCST	*Homo sapiens*	HE； M	10.1	3.4	161	220	14	Adenylate kinase isoenzyme 4,mitochondrial
**PPK2**	AK5/Q9Y6K8	AK5/Q9Y6K8	2bwj-A	+3.32	PCST	*Homo sapiens*	HE； M	10.8	3.5	160	196	9	Adenylate kinase isoenzyme 5

**PPK2**	ARL6/Q9H0F7*	ARL6/Q9H0F7*	2h57-A	+2.29e+04	ST	*Homo sapiens*	HE； M	3.9	3.6	98	165	13	ADP-ribosylation factor-like protein 6
**PPK2**	ASNA1/O43681	GET3/Q4WY07	3ibg-A	+9.67e+03	ST	*Aspergillus fumigatus*	LE	3.1	4.4	117	300	12	ATPase ASNA1
**PPK2**	ATP5A1/P25705*	ATP5A1/P19483*	2wss-A	+3.52e+04	PCST	*Bos taurus*	HE； M	2.4	4.1	114	510	10	ATP synthase subunit alpha,mitochondrial
**PPK2**	CACNB2/Q08289	CACNB2/P54288	1t3s-A	+60.09	PCST	*Oryctolagus cuniculus*	HE； M	8	9.3	183	294	9	Voltage-dependent L-type calcium channel subunit beta-2
**PPK2**	CACNB3/P54284	CACNB3/P54287	1vyt-A	+40.17	ST	*Rattus norvegicus*	HE； M	8.1	9.1	176	273	10	Voltage-dependent L-type calcium channel subunit beta-3
**PPK2**	CACNB4/O00305	CACNB4/Q8R0S4	1vyv-B	+8.53	ST	*Mus musculus*	HE； M	8	9.7	176	275	8	Voltage-dependent L-type calcium channel subunit beta-4
**PPK2**	CASK/O14936**	CASK/O14936**	1kgd-A	+195.56	ST	*Homo sapiens*	HE； M	6.9	4	136	175	7	Peripheral plasma membrane protein CASK
**PPK2**	CMPK1/P30085	PYRK/P20425	1uke-A	+841.29	PCST	*Dictyostelium discoideum*	LE	10.9	3.4	164	193	9	UMP-CMP kinase
**PPK2**	CMPK2/Q5EBM0**	TMK/Q970Q8**	2plr-B	+71.70	PCST	*Sulfolobus tokodaii*	P； A	12.6	3.2	164	208	15	UMP-CMP kinase 2,mitochondrial
**PPK2**	COASY/Q13057	COAE/Q56416**	1uf9-A	+6.49	ST,PC	*Thermus thermophilus*	P； B	6.6	3.6	134	191	10	Bifunctional coenzyme A synthase
**PPK2**	CTPS/P17812*,CTPS2/Q9NRF8*	PYRG/P0A7E5*	1s1m-B	+5.55e+03	ST	*Escherichia coli*	P； B	5.8	3.7	133	536	11	CTP synthase
**PPK2**	DCK/P27707*	DCK/P27707*	2a30-D	+6.63e+03	ST	*Homo sapiens*	HE； M	9.8	4.5	169	215	15	Deoxycytidine kinase
**PPK2**	DDX18/Q9NVP1	MSS116/P15424	3i5x-A	+994.16	ST,PC	*Saccharomyces cerevisiae*	LE	2.6	7.1	117	509	7	ATP-dependent RNA helicase DDX18
**PPK2**	DDX19B/Q9UMR2	DDX19B/Q9UMR2	3ews-A	+160.37	ST,PC	*Homo sapiens*	HE； M	2.2	4.8	97	416	9	ATP-dependent RNA helicase DDX19B
**PPK2**	DGUOK/Q16854**	DGUOK/Q93IG4	2jas-C	+615.99	ST,PC	*Mycoplasma mycoides*	P； B	9.6	3.3	151	195	15	Deoxyguanosine kinase,mitochondrial
**PPK2**	DLG4/P78352	DLG4/P31016	1jxm-A	+8.24e+03	PCST	*Rattus norvegicus*	HE； M	7.1	3.6	139	264	13	Disks large homolog 4
**PPK2**	DNM1/Q05193*	DNM1/Q05193*	2x2e-A	+1.68e+04	ST,PC	*Homo sapiens*	HE； M	2.4	3.8	110	341	9	Dynamin-1
**PPK2**	DTYMK/P23919**	DTYMK/P23919**	1e2d-A	+4.49e+03	ST,PC	*Homo sapiens*	HE； M	12.1	3	164	209	14	Thymidylate kinase
**PPK2**	EIF2S3/P41091	EIF2G/Q8U082	2dcu-A	+8.28e+03	ST,PC	*Pyrococcus furiosus*	P； A	3.5	4.4	108	407	11	Eukaryotic translation initiation factor 2 subunit 3
**PPK2**	EIF5B,MTIF2<br>O60841,P46199	INFB/P0A705	1zo1-I	+18.11	ST,PC	*Escherichia coli*	P； B	4	3.9	111	501	9	Eukaryotic translation initiation factor
**PPK2**	ERAL1/O75616	ERA/O67800	3iev-A	+5.07e+03	ST,PC	*Aquifex aeolicus*	P； B	3.1	3.9	95	302	12	GTPase Era,mitochondrial
**PPK2**	GTPBP3/Q969Y2*	MNME/Q9WYA4	1xzp-A	Unidentified	ST,PC	*Thermotoga maritima*	P； B	3.9	11.5	133	456	9	tRNA modification GTPase GTPBP3,mitochondrial
**PPK2**	GUK1/Q16774**	GUK1/P15454	1ex6-A	+4.63	PCST	*Saccharomyces cerevisiae*	LE	8.4	3.2	141	186	13	Guanylate kinase
**PPK2**	HRAS/P01112	HRAS/P01112	6q21-A	+2.55e+04	ST,PC	*Homo sapiens*	HE； M	3.7	4.2	97	171	14	GTPase HRas
**PPK2**	HS3ST1/O14792*	HS3ST1/O35310	1vkj-A	+414.82	PCST	*Mus musculus*	HE； M	6.4	4.1	143	258	9	Heparan sulfate glucosamine 3-O-sulfotransferase 1
**PPK2**	KHK/P50053	TM1040_0371*/Q1GJR2*	3c8u-A	+265.27	PCST	*Silicibacter sp.*	P； B	4.5	4.3	130	206	12	Ketohexokinase
**PPK2**	MMAA/Q8IVH4	MMAA/Q8IVH4	2www-D	+1.43e+03	ST,PC	*Homo sapiens*	HE； M	5	4.6	132	313	8	Methylmalonic aciduria type A protein,mitochondrial
**PPK2**	MYO5A/Q9Y4I1	MYO5A/Q02440	2dfs-A	-0.41	ST,PC	*Gallus gallus*	HE	2.3	4.9	126	994	11	Myosin-Va
**PPK2**	NDST1/P52848	NDST1/P52848	1nst-A	+7.24e+03	PCST	*Homo sapiens*	HE； M	5.3	3.8	135	282	5	Bifunctional heparan sulfate N-deacetylase/N-sulfotransferase 1
**PPK2**	NRK1/Q9NWW6	NRK1/Q9NWW6	2ql6-A	+6.23e+03	ST,PC	*Homo sapiens*	HE； M	9.8	3.1	143	179	9	Nicotinamide riboside kinase 1
**PPK2**	NUBP1/P53384,NUBP2/Q9Y5Y2	PF0485*/Q8U3I1	1g3r-A	+1.03e+04	ST	*Pyrococcus furiosus*	P； A	3.8	3.6	122	237	11	Cytosolic Fe-S cluster assembly factor
**PPK2**	PANK1/Q8TE04,PANK2/Q9BZ23,	COAA/P0A6I3	1esn-A	+2.73e+03	ST	*Escherichia coli*	P； B	5.9	4.1	155	311	8	Pantothenate kinase

	PANK3/Q9H999*,PANK4/Q9NVE7*												
**PPK2**	PAPSS1/O43252**	PAPSS1/O43252**	2ofw-A	+4.43e+04	ST	*Homo sapiens*	HE: M	7.9	3.5	154	200	9	Bifunctional 3'-phosphoadenosine 5'-phosphosulfate synthase 1
**PPK2**	PAPSS2/O95340**	SAT/CYSC/O67174*	2gks-A	-0.40	PCST	*Aquifex aeolicus*	P: B	7.8	8.6	200	522	11	Bifunctional 3'-phosphoadenosine 5'-phosphosulfate synthase 2
**PPK2**	PDHA1/P08559,PDHA2/P29803, PDHB/P11177	ACEE/P0AFG8*	2g25-A	+270.59	ST,PC	*Escherichia coli*	P: B	3.4	4.7	195	831	7	Pyruvate dehydrogenase E1 component,mitochondrial
**PPK2**	PFKFB1/P16118	F6PK/PFRX/P16118	1k6m-A	+251.82	PCST	*Homo sapiens*	HE: M	6.4	4	145	432	11	6-phosphofructo-2-kinase/fructose-2,6-biphosphatase 1
**PPK2**	PFKFB3/Q16875*	PFKFB3/Q16875*	2axn-A	+160.43	PCST	*Homo sapiens*	HE: M	6.5	4.7	143	451	13	6-phosphofructo-2-kinase/fructose-2,6-biphosphatase 3
**PPK2**	PFKFB4/Q16877	PFKFB4/P25114	1bif-A	+83.33	PCST	*Rattus norvegicus*	HE: M	6.8	3.9	144	432	10	6-phosphofructo-2-kinase/fructose-2,6-biphosphatase 4
**PPK2**	PMVK/Q15126	PMVK/Q15126	3ch4-B	Unidentified	PCST	*Homo sapiens*	HE: M	7.9	3.6	148	188	11	Phosphomevalonate kinase
**PPK2**	PNKP/Q96T60	PNKP/Q9JLV6	1yj5-A	+136.13	ST,PC	*Mus musculus*	HE: M	7.1	3.6	137	379	15	Bifunctional polynucleotide phosphatase/kinase
**PPK2**	PSTK/Q8IV42	PSTK/Q58933	3a4m-A	+79.97	ST,PC	*Methanocaldococcus jannaschii*	P: A	9.4	3.8	152	236	9	L-seryl-tRNA(Sec) kinase
**PPK2**	RAB11A/P62491**	YPT31/P38555**	3cpj-B	+2.46e+04	ST,PC	*Saccharomyces cerevisiae*	LE	2.7	5	108	164	7	Ras-related protein Rab-11A
**PPK2**	RAB27B/O00194	RAB27B/Q99P58	2zet-A	+8.59e+03	ST	*Mus musculus*	HE: M	4.6	4	102	181	10	Ras-related protein Rab-27B
**PPK2**	RAB6A/P20340**	RAB6A/P20340**	3cwz-A	+1.29e+04	ST,PC	*Homo sapiens*	HE: M	4.3	3.6	98	166	8	Ras-related protein Rab-6A
**PPK2**	RAC3/P60763	ARAC2/Q38903	2wbl-D	+1.36e+04	ST,PC	*Arabidopsis thaliana*	HE: P	3.8	3.6	93	168	10	Ras-related C3 botulinum toxin substrate 3
**PPK2**	RECQL/P46063	RECQL/P46063	2wwy-A	+5.10	ST,PC	*Homo sapiens*	HE: M	2.8	5.5	115	530	8	ATP-dependent DNA helicase Q1
**PPK2**	RERG/Q96A58*	RERG/Q96A58*	2atv-A	+1.92e+04	ST	*Homo sapiens*	HE: M	4.5	4	108	168	12	Ras-related and estrogen-regulated growth inhibitor
**PPK2**	RHOA/P61586	RHOA/P61586	1cxz-A	+1.56e+04	ST,PC	*Homo sapiens*	HE: M	3.5	3.5	97	182	14	Transforming protein RhoA
**PPK2**	RRAS/P10301	RRAS/P10301	2fn4-A	+2.07e+04	ST	*Homo sapiens*	HE: M	4	3.8	101	173	14	Ras-related protein R-Ras
**PPK2**	SEPT2/Q15019	SEPT2/P42208*	3ftq-A	+1.84e+04	ST	*Mus musculus*	HE: M	5.2	4.1	116	254	13	Septin-2
**PPK2**	SRP54/P61011**	SRP54/Q57565**	2v3c-C	+0.27	ST,PC	*Methanocaldococcus jannaschii*	P: A	3.2	17.2	135	403	5	Signal recognition particle 54 kDa protein
**PPK2**	SULT1A1/P50225*	SULT1A1/P50225*	1z28-A	+9.11e+03	ST,PC	*Homo sapiens*	HE: M	4.7	4.6	141	289	7	Sulfotransferase 1A1
**PPK2**	SULT1A3/SULT1A4/P50224*	SULT1A3/SULT1A4/P50224*	1cjm-A	+8.27e+03	PCST	*Homo sapiens*	HE: M	5.1	4.8	138	223	8	Sulfotransferase 1A3/1A4
**PPK2**	SULT1B1/O43704	SULT1B1/O43704	2z5f-A	+2.08e+03	ST,PC	*Homo sapiens*	HE: M	4.6	4.5	142	293	8	Sulfotransferase family cytosolic 1B member 1
**PPK2**	SULT1C4/O75897	SULT1C4/O75897	2ad1-A	+8.99e+03	PCST	*Homo sapiens*	HE: M	4.8	4.2	134	244	6	Sulfotransferase 1C4
**PPK2**	TAF9/Q9Y3D8	TAF9/Q9Y3D8	1rkb-A	+3.73e+04	PCST	*Homo sapiens*	HE: M	9.4	3.5	153	173	12	Adenylate kinase isoenzyme 6
**PPK2**	TJP1/Q07157	TJP1/Q07157	3lh5-A	+27.87	PCST	*Homo sapiens*	HE: M	7.3	7.2	157	248	9	Tight junction protein ZO-1
**PPK2**	TJP3/O95049	TJP3/O95049	3kfv-A	+66.73	PCST	*Homo sapiens*	HE: M	7.7	5.4	158	241	9	Tight junction protein ZO-3
**PPK2**	TK1/P04183*,TK2/O00142*	TK/P03176	1e2h-A	+1.80e+04	ST,PC	*Human herpesvirus 1*	Virus	9.2	3.6	164	306	12	Thymidine kinase
**PPK2**	TRIT1/Q9H3H1	IPT/Q5GHF7*	3a8t-A	+252.11	PCST	*Humulus lupulus*	HE: P	8.3	3.5	154	289	6	tRNA dimethylallyltransferase,mitochondrial
**PPK2**	UCK1/Q9HA47*	UCK1/Q9HA47*	2jeo-A	+82.39	PCST	*Homo sapiens*	HE: M	6.8	3.9	144	212	10	Uridine-cytidine kinase 1
**PPK2**	UCK2/Q9BZX2*	UCK2/Q9BZX2*	1ufq-D	+21.43	PCST	*Homo sapiens*	HE: M	6.6	4	141	210	12	Uridine-cytidine kinase 2
**PPK2**	VCP/P55072	VCP/Q01853	3cf1-B	+35.77	ST,PC	*Mus musculus*	HE: M	4	4.1	128	723	10	Transitional endoplasmic reticulum ATPase

**PPK4**	ACYP1/P07311,ACYP2/P14621	ACYP/Q97ZL0*	1y9o-A	Unidentified	ST	*Sulfolobus solfataricus*	P; A	2.4	3.8	67	103	10	Acylphosphatase
**PPK4**	AGPS/O00116	EAPA/O96759	2uuu-B	+26.94	ST	*Dictyostelium discoideum*	LE	5.7	5.3	86	540	5	Alkyldihydroxyacetonephosphate synthase,peroxisomal
**PPK4**	ALLC/Q8N6M5	ALLC/P77425	1z2l-A	+22.66	ST	*Escherichia coli*	P; B	4.6	4.2	82	411	4	Allantoate amidinohydrolase
**PPK4**	AMD1/P17707	SPEH/Q9WZC3*	1tlu-A	+1.58e+04	ST	*Thermotoga maritima*	P; B	5.2	3	78	117	12	S-adenosylmethionine decarboxylase proenzyme
**PPK4**	AMT/P48728	GCVT/P27248*	1vlo-A	+23.38	ST	*Escherichia coli*	P; B	2.1	3.4	64	364	11	Aminomethyltransferase,mitochondrial
**PPK4**	AP2A2/O94973	AP2A2/P17427	1kyf-A	+1.20e+03	ST	*Mus musculus*	HE; M	3.3	3.8	78	247	12	AP-2 complex subunit alpha-2
**PPK4**	ARPC2/O15144	ARC2/O14241	3dwl-D	+1.16e+03	ST,PC	*Schizosaccharomyces pombe*	LE	2.7	3.8	64	272	11	Actin-related protein 2/3 complex subunit 2
**PPK4**	ATOX1/O00244	ATX1/P38636	1cc8-A	+4.57e+03	ST,PC	*Saccharomyces cerevisiae*	LE	3.5	3.5	63	72	13	Copper transport protein ATOX1
**PPK4**	ATP7A/Q04656	ATP7A/Q04656	1yjv-A	+4.31e+03	ST	*Homo sapiens*	HE; M	2.7	3.8	64	75	9	Copper-transporting ATPase 1
**PPK4**	CNDP2/Q96KP4	CNDP2/Q9D1A2	2zof-A	Unidentified	ST	*Mus musculus*	HE; M	2.7	3.8	79	478	8	Cytosolic non-specific dipeptidase
**PPK4**	COPG/Q9Y678*	COPG/Q9Y678*	1r4x-A	+6.60	ST	*Homo sapiens*	HE; M	3.9	3.5	77	274	13	Coatomer subunit gamma
**PPK4**	PM20D1/Q6GTS8	CPG2/P06621	1cg2-A	+12.65	ST,PC	*Pseudomonas sp.*	P; B	3.8	4.2	78	389	9	Carboxypeptidase
**PPK4**	CPT2/P23786	CPT2/P18886	2deb-A	+109.67	ST	*Rattus norvegicus*	HE; M	2	3.8	69	627	12	Carnitine O-palmitoyltransferase 2,mitochondrial
**PPK4**	CUTA/O60888	CUTA/Q9X0E6	1kr4-A	+2.53e+04	ST	*Thermotoga maritima*	P; B	3.2	5.4	75	110	8	Divalent-cation tolerance protein CutA
**PPK4**	EEF1B2/P24534	EFB1/P32471*	1g7c-B	+1.04e+04	ST	*Saccharomyces cerevisiae*	LE	3.6	3.9	72	90	7	Elongation factor 1-beta
**PPK4**	EEF2/P13639*	EFT1/EFT2/P32324	1zm9-E	+43.41	ST	*Saccharomyces cerevisiae*	LE	2.7	6.5	72	823	10	Elongation factor 2
**PPK4**	EIF2A/Q9BY44	EIF2A/Q9V0E4	1yz6-A	+4.13	ST,PC	*Pyrococcus abyssi*	P; A	4.7	3.7	74	261	7	Eukaryotic translation initiation factor 2A
**PPK4**	EIF2S1/P05198	EIF2S1/P05198	1q8k-A	-0.25	PCST	*Homo sapiens*	HE; M	3	4.8	79	300	10	Eukaryotic translation initiation factor 2 subunit 1
**PPK4**	GCLC/P48506	GSH1/O23736	2gwc-F	+190.50	ST	*Brassica juncea*	HE; P	2.2	8.1	96	440	11	Glutamate--cysteine ligase catalytic subunit
**PPK4**	GFM1/Q96RP9,GFM2/Q969S9	FUSA/P13551*	2bm0-A	+271.79	ST	*Thermus thermophilus*	P; B	3.8	6.8	75	666	8	Elongation factor G,mitochondrial
**PPK4**	GUF1/Q8N442*	LEPA/O67618	2ywe-A	+3.92	ST,PC	*Aquifex aeolicus*	P; B	3.6	5.7	79	526	10	Translation factor GUF1,mitochondrial
**PPK4**	HMGCR/P04035	HMGCR/P04035	3cct-C	+90.06	PCST	*Homo sapiens*	HE; M	3.5	6.4	70	417	11	3-hydroxy-3-methylglutaryl-coenzyme A reductase
**PPK4**	HMOX1/P09601*,HMOX2/P30519	ISDG/Q8NX62*	1xbw-A	Unidentified	ST,PC	*Staphylococcus aureus*	P; B	3.5	3.9	70	99	1	Heme oxygenase
**PPK4**	HNRNPK/P61978	HNRNPK/P61978	1zzk-A	+9.48e+03	PCST	*Homo sapiens*	HE; M	3.4	2.9	62	80	11	Heterogeneous nuclear ribonucleoprotein K
**PPK4**	KHSRP/Q92945	KHSRP/Q92945	2opu-A	+1.18e+04	ST	*Homo sapiens*	HE; M	2.3	3.5	57	89	14	Far upstream element-binding protein 2
**PPK4**	KIAA0907/Q7Z7F0	KIAA0907/Q7Z7F0	2yqr-A	+4.10e+03	Unidentified	*Homo sapiens*	HE; M	2.6	9.1	72	119	8	UPF0469 protein KIAA0907
**PPK4**	MED18/Q9BUE0	MED18/O14198	3c0t-A	+29.63	ST	*Schizosaccharomyces pombe*	LE	8.6	3.9	160	201	9	Mediator of RNA polymerase II transcription subunit 18
**PPK4**	MED20/Q9H944	SRB2/P34162	2hzm-A	+1.19e+04	ST,PC	*Saccharomyces cerevisiae*	LE	6.4	4.8	148	205	11	Mediator of RNA polymerase II transcription subunit 20
**PPK4**	MESDC2/Q14696*	MESDC2/Q9ERE7*	2rqk-A	+2.40e+04	ST	*Mus musculus*	HE; M	3.3	3.3	63	109	11	LDLR chaperone MESD
**PPK4**	MPG/P29372	ALKA/P04395	3cvt-A	+5.46e+03	ST,PC	*Escherichia coli*	P; B	2.1	4.2	61	282	10	DNA-3-methyladenine glycosylase
**PPK4**	MT-CYB/P00156	MT-CYB/P18946	2bcc-B	+0.71	ST,PC	*Gallus gallus*	HE	2.8	3.9	74	406	7	Cytochrome b
**PPK4**	NOVA1/P51513*	NOVA1/P51513*	2anr-A	+1.45e+04	ST,PC	*Homo sapiens*	HE; M	3.9	5.6	79	155	9	RNA-binding protein Nova-1
**PPK4**	NUAK2/Q9H093	ATSR1/Q9LZW4	2zfd-B	+9.35e+03	PCST	*Arabidopsis thaliana*	HE; P	4.4	3.5	77	116	8	NUAK family SNF1-like kinase 2

**PPK4**	PAEP/P09466	BLG/LOC396596*/P04119	1exs-A	+1.08e+04	ST	*Sus scrofa*	HE； M	2.1	4.4	72	160	10	Glycodelin
**PPK4**	PAH/P00439*	PAH/P04176	1phz-A	+1.86e+03	ST	*Rattus norvegicus*	HE； M	3.9	3.4	73	403	5	Phenylalanine-4-hydroxylase
**PPK4**	PCBD1/P61457*	PCD/Q2Q449**	2v6u-A	+7.29e+03	ST,PC	*Toxoplasma gondii*	LE	3.2	3.5	64	103	9	Pterin-4-alpha-carbinolamine dehydratase
**PPK4**	PCBD2/Q9H0N5	PCBD2/Q9CZL5	1ru0-A	+2.20e+04	ST,PC	*Mus musculus*	HE； M	3	3.7	62	100	6	Pterin-4-alpha-carbinolamine dehydratase 2
**PPK4**	PCBP1/Q15365*	PCBP1/Q15365*	1wvn-A	+7.13e+03	ST,PC	*Homo sapiens*	HE； M	2.7	2.9	57	74	11	Poly(rC)-binding protein 1
**PPK4**	PCBP2/Q15366*	PCBP2/Q15366*	2pqu-A	+912.90	ST,PC	*Homo sapiens*	HE； M	4.1	3.1	63	72	13	Poly(rC)-binding protein 2
**PPK4**	PET112L/O75879	GATB/O66766	3h0l-B	+8.08e+03	ST	*Aquifex aeolicus*	P； B	2.7	5.5	108	410	8	Glutamyl-tRNA(Gln) amidotransferase subunit B,mitochondrial
**PPK4**	PGM1/P36871	PP63-1/P47244	1kfi-A	+547.43	ST	*Paramecium tetraurelia*	LE	6.3	3.4	92	570	8	Phosphoglucomutase-1
**PPK4**	PHGDH/O43175*	SERA/P0A544	1ygy-A	+89.92	ST	*Mycobacterium tuberculosis*	P； B	2.8	3.6	64	527	13	D-3-phosphoglycerate dehydrogenase
**PPK4**	PMPCB/O75439*	MAS1/P10507	1hr9-F	+1.28	ST,PC	*Saccharomyces cerevisiae*	LE	2.2	5.6	75	443	5	Mitochondrial-processing peptidase subunit beta
**PPK4**	Rcan1/P53805	RCAN1/Q9JHG6	1wey-A	+2.89	ST,PC	*Mus musculus*	HE； M	2.2	4	64	104	5	Calcipressin-1
**PPK4**	RPIA/P49247	RPIA/Q72J47*	1uj4-A	+1.25e+03	ST,PC	*Thermus thermophilus*	P； B	2.9	4.2	64	225	9	Ribose-5-phosphate isomerase
**PPK4**	SMS/P52788	SMS/P52788	3c6k	+71.17	ST	*Homo sapiens*	HE； M	5.5	5.1	87	348	5	Spermine synthase
**PPK4**	STARD4/Q96DR4	STARD4/Q99JV5	1jss-A	+256.40	ST,PC	*Mus musculus*	HE； M	3.1	3.6	79	199	8	StAR-related lipid transfer protein 4
**PPK4**	TBP/P20226**	SPT15/P13393	1rm1-A	+0.80	PC	*Saccharomyces cerevisiae*	LE	3	3.6	66	180	5	TATA-box-binding protein
**PPK4**	THTPA/Q9BU02*	THTPA/Q8JZL3*	2jmu-A	+4.63	ST	*Mus musculus*	HE； M	5.4	4.8	145	224	16	Thiamine-triphosphatase
**PPK4**	TOP2A/P11388,TOP2B/Q02880	GYRA/P0AES4	2y3p-B	+1.28e+04	ST	*Escherichia coli*	P； B	2.1	6.2	68	506	7	DNA topoisomerase 2
**PPK4**	UPB1/Q9UBR1	PYD3/Q96W94	2v8g-C	+1.21e+03	ST	*Saccharomyces kluyveri*	LE	3.9	4.4	81	432	10	Beta-ureidopropionase
**PPK4**	UQCRC1/P31930	UQCRC1/P31800*	1l0l-A	+0.71	ST	*Bos taurus*	HE； M	3	3.4	76	446	4	Cytochrome b-c1 complex subunit 1,mitochondrial
