# Supplementary material for: Positively-Charged Semi-Tunnel Is a Structural and Surface Characteristic of Polyphosphate-Binding Proteins: An In-Silico Study
Source: PLoS One. 2015 Apr 16;10(4):e0123713. doi: 10.1371/journal.pone.0123713 (PMC4400040; doi:10.1371/journal.pone.0123713)
Supplement: S1 Text — (PDF) [file pone.0123713.s002.pdf]

Utilizing the Dali program to search the Protein Data Bank (PDB) protein structures, we found 2,247 structure chains with partial similarity to three reported PPKs (687 chains were similar to PPK1 from *Escherichia coli*, 812 chains were similar to PPK2 from *Pseudomonas aeruginosa* and 748 chains were similar to PPK4 from *Saccharomyces cerevisiae*). After manually removing redundant entries, 242 structure chains and their genes were finally identified. We designated these proteins “partial structural analogs of PPKs”, and predicted that some of them retained characteristics of polyP-related proteins. Nine of the 242 structure chains yielded errors when performing molecular docking calculations.
